# The *Caenorhabditis elegans* T-Box Factor MLS-1 Requires Groucho Co-Repressor Interaction for Uterine Muscle Specification

**DOI:** 10.1371/journal.pgen.1002210

**Published:** 2011-08-11

**Authors:** Raymond R. Miller, Peter G. Okkema

**Affiliations:** Department of Biological Sciences, Laboratory for Molecular Biology, University of Illinois at Chicago, Chicago, Illinois, United States of America; University of California San Diego, United States of America

## Abstract

T-box proteins are conserved transcription factors that play crucial roles in development of all metazoans; and, in humans, mutations affecting T-box genes are associated with a variety of congenital diseases and cancers. Despite the importance of this transcription factor family, very little is known regarding how T-box factors regulate gene expression. The *Caenorhabditis elegans* genome contains 21 T-box genes, and their characterized functions include cell fate specification in a variety of tissues. The *C. elegans* Tbx1 sub-family member MLS-1 functions during larval development to specify the fate of non-striated uterine muscles; and, in *mls-1* mutants, uterine muscles are transformed to a vulval muscle fate. Here we demonstrate that MLS-1 function depends on binding to the Groucho-family co-repressor UNC-37. MLS-1 interacts with UNC-37 via a conserved eh1 motif, and the MLS-1 eh1 motif is necessary for MLS-1 to specify uterine muscle fate. Moreover, *unc-37* loss-of-function produces uterine muscle to vulval muscle fate transformation similar to those observed in *mls-1* mutants. Based on these results, we conclude that MLS-1 specifies uterine muscle fate by repressing target gene expression, and this function depends on interaction with UNC-37. Moreover, we suggest that MLS-1 shares a common mechanism for transcriptional repression with related T-box factors in other animal phyla.

## Author Summary

T-box proteins are a conserved family of transcription factors that play important roles in development of all animals. A variety of human congenital diseases and cancers have been associated with mutations that either decrease or increase T-box factor activity. By learning more about how T-box transcription factors function, we can better understand and treat these disorders. Here we use genetic and molecular techniques in the roundworm *C. elegans* to explore the mechanism(s) of the muscle-specifying T-box factor MLS-1. We show that MLS-1 physically interacts with another conserved protein called Groucho to repress gene expression and that this interaction is necessary for normal MLS-1 function. C. *elegans* MLS-1 is highly related to T-box factors in humans and other organisms, and we suggest Groucho interaction may be a common mechanism for T-box factor function. These insights may lead to therapies that modulate the activity of T-box factors in disease.

## Introduction

T-box transcription factors play essential roles in the development of all multicellular organisms, where their functions include the specification of primary germ layers and the specification of cell fates during organogenesis [1], [2]. In humans, both decreased and increased activity of these factors are associated with congenital disease (Holt-Oram syndrome, Ulnar-Mammary syndrome, DiGeorge syndrome, etc) [3], auto immune disorders [4], and cancers [5], [6]. Despite this importance the mechanisms by which T-box factors regulate target gene expression are not well established.

Groucho family (Gro/TLE) proteins are conserved transcriptional co-repressors that interact with distinct Engrailed homology 1 (eh1) or WRPW/Y motifs in a wide variety of transcription factors and, in many cases, recruit histone deacetylases to target gene promoters [reviewed in [7]]. Gro/TLE factors have recently been implicated in the regulatory mechanism of several T-box factors. Xenopus Tbx6 and Tbx1, and zebrafish Tbx24 and Ntl/Brachyury interact indirectly with Gro/TLE factors through Ripply/Bowline family proteins, and this interaction can convert these proteins from transcriptional activators to repressors [8]–[10]. Two closely related members of the mouse Tbx1 subfamily Tbx15 and Tbx18 interact directly with the Gro/TLE protein TLE3 via eh1 motifs to repress reporter gene expression in mammalian cells [11]. Additional T-box factors likely function with Gro/TLE proteins, as T-box factors in several species contain eh1 motifs, including the *Caenorhabditis elegans* T-box factors MLS-1 and MAB-9 [12]. While this accumulating evidence suggests a variety of T-box factors interact with Gro/TLE factors, the significance of these interactions has not been examined *in vivo*.

In this report we investigate the interaction between MLS-1 and the *C. elegans* Gro/TLE protein UNC-37. MLS-1 is a member of the Tbx1 subfamily that includes mouse Gro/TLE-interacting proteins Tbx15 and Tbx18 [1], [13]. MLS-1 functions to specify uterine muscle fate in the mesodermal (M) lineage during hermaphrodite larval development [14]. In wild-type hermaphrodites, the M mesoblast produces all post-embryonic mesoderm cells, including two sex myoblasts (SMs) that divide during the late L3 and L4 stages to produce eight uterine muscles (four um1 and four um2 uterine muscles) and eight vulval muscles (four vm1 and four vm2 vulval muscles) [15]. *mls-1* loss-of-function results in a transformation of uterine muscle precursors to a vulval muscle fate resulting in the loss of all um1 and um2 muscles and the formation of excess vm1 and vm2 muscles. In comparison, ectopic expression of *mls-1* throughout the M lineage results in supernumerary uterine muscles [14].

Here we demonstrate that MLS-1 interacts with UNC-37 in both yeast two-hybrid and in *C. elegans* bimolecular fluorescence complementation (BiFC) assays. This interaction is mediated by an eh1 motif near the MLS-1 N-terminus, and mutation of this eh1 motif eliminates the ability of MLS-1 to specify uterine muscles. Furthermore, *unc-37* loss-of-function results in a loss of uterine muscles and a corresponding gain of vulval muscles similar to *mls-1* loss-of-function. Taken together, these results indicate MLS-1 functions as an UNC-37 dependent transcriptional repressor to specify uterine muscle fate, and they provide the first *in vivo* evidence that interaction with Gro/TLE factors is essential for T-box factor function.

## Results

### MLS-1 and UNC-37 interact in both yeast and worms via a conserved eh1 motif

**Figure 1:**
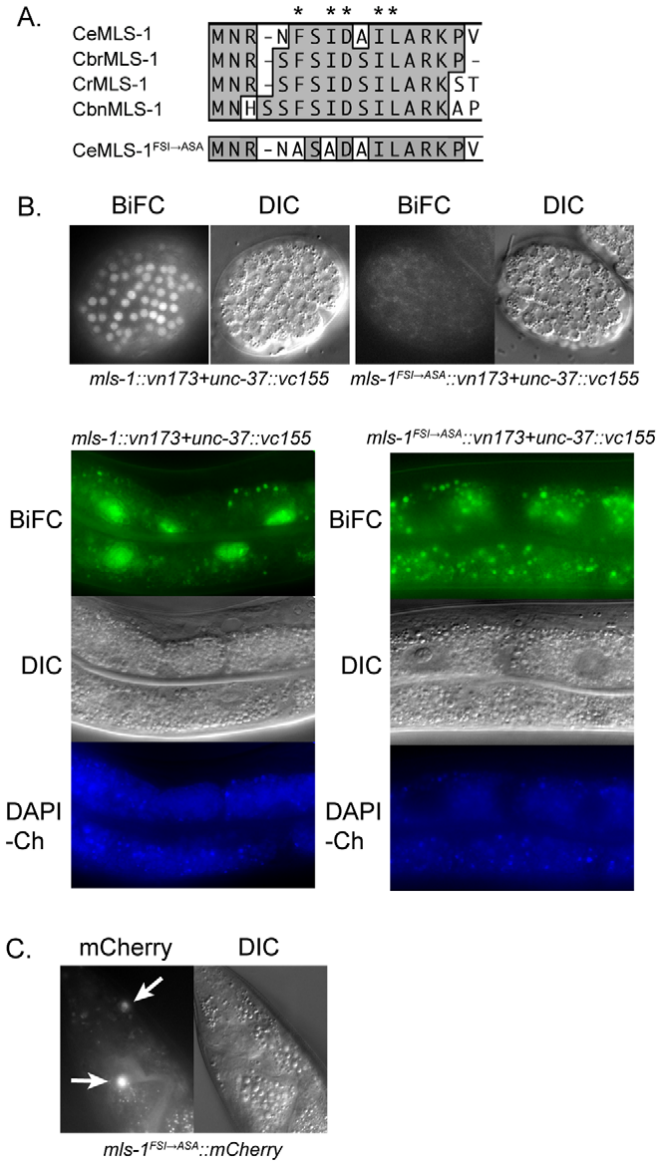
MLS-1 and UNC-37 physically interact in an MLS-1 eh1 motif-dependent manner. A) ClustalW alignment of the N-terminus of *C. elegans* (Ce), *C. briggsae* (Cbr), *C. remanei* (Cr), *C. brenneri* (Cbn), and mutated *C. elegans* MLS-1 sequences. Asterisks are above eh1 motif core residues. Two of these residues in *C. elegans* MLS-1 were mutated to alanines producing *C. elegans* MLS-1^FSI→ASA^. B) Transgenic animals (OK0691) co-expressing *mls-1::vn173* and *unc-37::vc155* (left column) show BiFC signal in embryo (top) and adult intestinal (bottom) nuclei. Animals (OK0710) co-expressing *mls-1^FSI→ASA^::vn173* and *unc-37::vc155* (right column) show no BiFC signal. Transgene expression is controlled by *hsp-16.4* promoter. Gut granule autofluorescence is shown by images of DAPI channel (DAPI-Ch). C) MLS-1^FSI→ASA^::mCherry fusion proteins expressed using the *mls-1* promoter are observed in enteric muscles (arrows).

*C. elegans* MLS-1 is a relatively small T-box protein (252 aa; Accession NP_498640) consisting of a 187 residue T-box DNA-binding domain flanked by short N-terminal and C-terminal amino acid stretches [14]. A bioinformatic screen of predicted transcription factors in *C. elegans* identified a high-scoring eh1 motif outside of the T-box near the MLS-1 N-terminus [12]. The MLS-1 eh1 motif is conserved in the N-terminus of MLS-1 proteins of several *Caenorhabditis* species (Figure 1A) suggesting it is biologically significant, and we hypothesize MLS-1 functions as a Groucho-dependent transcriptional repressor.

To test this hypothesis, we first asked if MLS-1 can interact with the *C. elegans* Groucho protein UNC-37 using yeast two-hybrid assays. A full-length MLS-1 bait was tested for interaction with a prey encoding UNC-37 residues 70–612, which consists of the entire WD repeat domain (Accession O02482). The WD repeat domain of Gro/TLE proteins mediates interaction with target proteins [7], [16], [17]. We found that MLS-1 interacts specifically with the UNC-37 prey, but does not interact with the empty prey vector. Mutating two consensus residues in the MLS-1 eh1 motif to alanines abolished this interaction (Figure 1A). In subsequent experiments we refer to this mutation as MLS-1^FSI→ASA^.

To further explore this physical interaction we asked if MLS-1 and UNC-37 are able to undergo bimolecular fluorescence complementation (BiFC) in *C. elegans*. In BiFC assays separate pieces of a fluorescent protein are attached to potential protein interactors, and if these proteins interact the fluorescent protein is able to reconstitute giving off a fluorescent signal [18], [19]. We generated three independent transgenic lines expressing *mls-1* fused to a Venus N-terminal fragment (*mls-1::vn173*) and *unc-37* fused to a Venus C-terminal fragment (*unc-37::vc155*) under control of the heat shock promoter *hsp-16.41*. Heat shocking each of these lines resulted in BiFC signal in the nuclei of several cell types in which the *hsp-16.41* promoter is strongly active after heat shock, most noticeably in nuclei in embryos and the intestinal cells (Figure 1B) [20]. The nuclear BiFC signal in the intestine was distinct from autofluorescence from endogenous gut granules (Figure 1B), and transgenic lines expressing only unfused Venus protein halves had no BiFC signal as previously reported [18].

To determine whether the interaction between MLS-1 and UNC-37 requires the MLS-1 eh1 motif, we asked whether co-expression of *mls-1^FSI→ASA^::vn173* and *unc-37::vc155* would present a BiFC signal. After a one hour heat shock, transgenic animals co-expressing these proteins displayed no BiFC signal, whereas animals co-expressing wild-type *mls-1::vn173* with *unc-37::vc155* exhibited abundant BiFC signal (Figure 1B). Substantially longer heat shocks caused some nuclei co-expressing *mls-1^FSI→ASA^::vn173* and *unc-37::vn155* to exhibit low intensity BiFC signal, suggesting that the eh1 mutation reduced but did not completely eliminate interaction with UNC-37, and that the MLS-1^FSI→ASA^ protein is expressed. To further demonstrate that the MLS-1^FSI→ASA^ protein is expressed, we examined strains containing an *mls-1^FSI→ASA^::mCherry* transgene regulated by the *mls-1* promoter, and found that this mutant fusion protein is expressed in enteric muscles at a level comparable to wild-type *mls-1::mCherry* (Figure 1C).

Taken together, these results indicate that MLS-1 can interact with UNC-37 in yeast and in *C. elegans*, and that these interactions depend on the MLS-1 eh1 motif. Furthermore, in *C. elegans* this interaction was detected only in cell nuclei, consistent with the hypothesis that these proteins interact to repress target gene expression.

### MLS-1 and UNC-37 interact in biologically relevant cell types

MLS-1 and UNC-37 are capable of interacting in yeast and when expressed broadly in *C. elegans* using a heat shock promoter, but it is not clear from these results whether these proteins interact in any of the cells in which MLS-1 functions. UNC-37 is believed to be ubiquitously expressed throughout development [17], but MLS-1 is expressed in a very restricted pattern in uterine muscles, type 2 vulval muscles (after specification of these cells as vulval muscles), and three enteric muscles (left and right intestinal muscles and anal depressor muscle) [14]. Our analysis of *mls-1* promoter activity confirms that *mls-1* is expressed in the above mentioned enteric muscles throughout the life of animals post-hatching, but is only very transiently expressed in the uterine muscles and vm2s at L4 and young adult stages (Figure S1).

**Figure 2:**
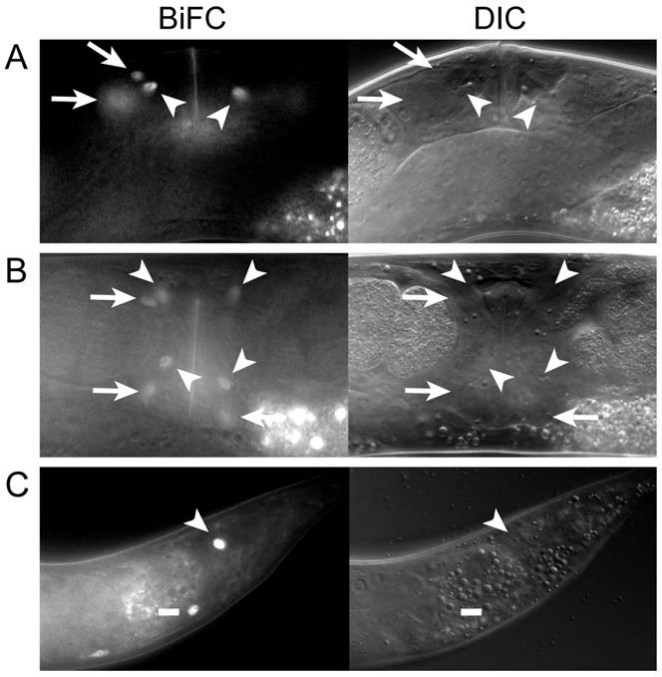
MLS-1 and UNC-37 are both co-expressed and interact in uterine, vulval, and enteric muscles. A, B) Lateral (A) and ventral (B) views of OK0742 young adult hermaphrodites co-expressing *mls-1::vn173* and *unc-37::vc155* under control of their own promoters exhibiting BiFC signal in uterine muscle (arrows) and vulval muscle (arrowheads) nuclei. Uterine muscle cytoplasm and nuclei displayed BiFC (compare arrows). C) Adult (OK0744) animal exhibiting BiFC in one intestinal muscle (dash) and the anal depressor (arrowhead).

To determine if MLS-1 and UNC-37 interact in cells normally expressing MLS-1, we generated three independent strains co-expressing *mls-1::vc155* and *unc-37::vn155* under control of the *mls-1* and *unc-37* promoters, respectively, and found that each of these lines exhibited BiFC signal in most cells where *mls-1* is normally expressed. BiFC signal was observed postembryonically in one or two cells in the tail, which we tentatively identify as enteric muscles (Figure 2C). BiFC signal was also observed near the vulva in young adults, in cells that we identify as the vm2 vulval muscles and the um1 and um2 uterine muscles based on position and morphology (Figure 2A, 2B). The um2 cells had BiFC signal localized in either nuclei or cytoplasm, and we believe these differences reflect the different morphology of type 1 and type 2 uterine muscles [21]. The signals in the vulval and uterine muscles were very transient and were not observed in L4s or in older adults.

### The MLS-1 eh1 motif is necessary for uterine muscle specification

**Figure 3:**
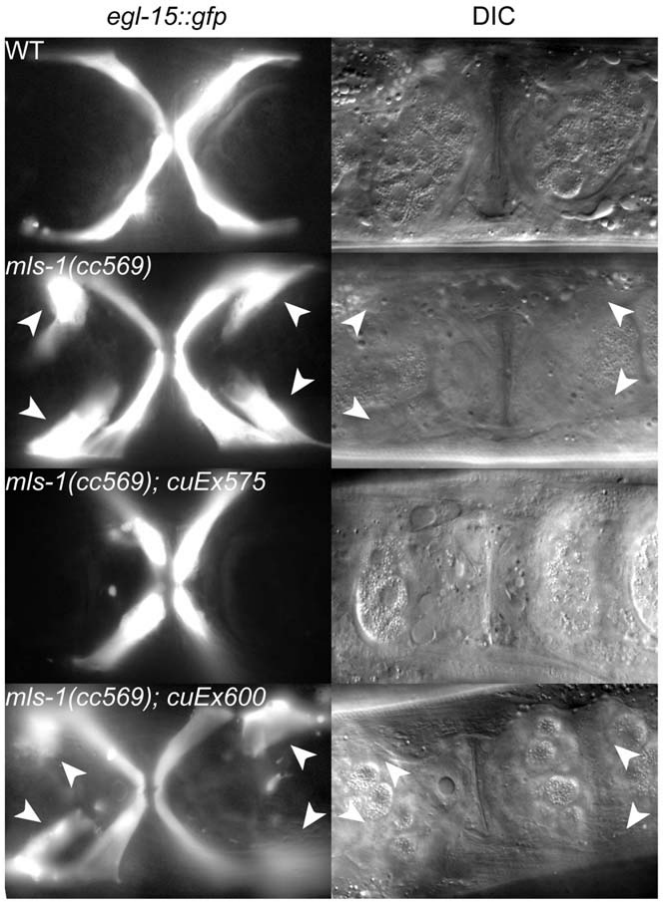
MLS-1 uterine muscle specification activity depends on its eh1 motif. Ventral view of wild-type (WT) and mutant animals of the indicated genotypes expressing *egl-15::gfp* in vm1 cells. The transgene *cuEx575* contains wild-type *mls-1*, while *cuEx600* contains *mls-1^FSI→ASA^*. Supernumerary vm1 cells (arrowheads) are present in *mls-1(cc569)* and *mls-1(cc569); cuEx600[mls-1^FSI→ASA^]*.

MLS-1 promotes uterine muscle fate, and we wanted to determine if this activity depends on the MLS-1 eh1 motif. In *mls-1* mutants uterine muscles are transformed into vulval muscles. This phenotype can be easily scored using an *egl-15::gfp* reporter, which in wild-type animals is expressed in four vm1 cells that form a characteristic X-shape near the vulva [22]–[24], but in *mls-1* mutants *egl-15::gfp* expression can be seen in four additional cells [14] (Figure 3). *mls-1* mutants can be efficiently rescued by transformation with a wild-type *mls-1* genomic DNA fragment injected at 10 ng/µl [14]. We wanted to ask if MLS-1 requires an intact eh1 motif to specify uterine muscle fate, but we were unable to obtain transgenic lines containing the same genomic fragment bearing the *mls-1^FSI→ASA^* mutation when injected at this concentration. This result strongly suggests that the *mls-1^FSI→ASA^* is expressed, but is toxic to the animals.

We were able to generate both wild-type *mls-1* and *mls-1^FSI→ASA^* transgenic strains by reducing the DNA concentration to 2.5 ng/µl. Wild-type *mls-1* DNA at this lower concentration was able to rescue *mls-1(cc569)* mutants in five independent lines, but at reduced percentages (Figure 3) (Table 1). In contrast the *mls-1^FSI→ASA^* transgene did not rescue *mls-1(cc569)* in seven of seven lines (Figure 3) (Table 1). These results indicate that the MLS-1 eh1 motif is required for MLS-1 to specify uterine muscle fate.

**Table 1:** Percentage of *mls-1(cc569)* animals rescued with *mls-1* and *mls-1^FSI→ASA^* transgenes.

Genotype^1^	Animals with wild-type *egl-15::gfp* expression
+/+	98% (n = 61)
*mls-1(cc569)*	0% (n = 56)
*mls-1(cc569); cuEx575 [mls-1]* 2	61% (n = 41)
*mls-1(cc569); cuEx597 [mls-1^FSI→ASA^]* 2	0% (n = 62)

*egl-15::gfp* expression in the vm1 vulval muscles was used to score the *mls-1* phenotype. *egl-15::gfp* expression is occasionally mosaic in wild-type and mutant animals. Animals containing four *egl-15::gfp* expressing vm1 vulval muscles were scored as wild type, while animals containing five to eight *egl-15::gfp* expressing vm1 cells were scored as mutant.

1 All genotypes include the *ayIs2[egl-15::gfp]* transgene.

2 Transgenic lines shown are representative of multiple independently generated lines.

### *unc-37* loss-of-function animals show sex muscle and mesoderm lineage defects

**Figure 4:**
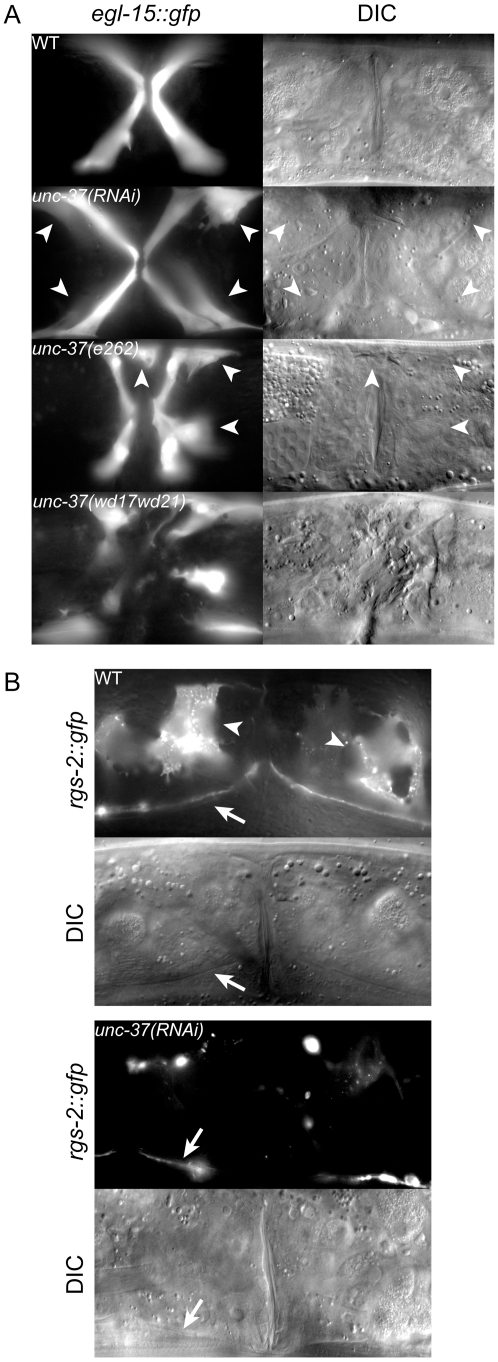
*unc-37* loss of function animals phenocopy *mls-1(cc569)*. A) Ventral view of wild-type (WT) and mutant animals of the indicated genotypes expressing *egl-15::gfp* in vm1 cells. Supernumerary vm1 cells (arrowheads) are present in *unc-37(RNAi)*, *unc-37(e262)*, and *unc-37(wd17wd21)* animals. *unc-37(wd17wd21)* mutants exhibit a protruding vulva phenotype with mispositioned vulval muscles, so the supernumerary muscles are not marked. B) Ventral views of wild-type (WT) and mutant animals of the indicated genotypes expressing *rgs-2::gfp*. In wild type, *rgs-2::gfp* is detected in uterine muscles (arrowheads), which appear as sheet-like muscles and in the ventral nerve cord (arrow). *unc-37(RNAi)* causes a decrease in number of uterine muscles cells expressing *rgs-2::gfp* and decreased fluorescence intensity in these cells.

Because the MLS-1 eh1 motif mediates interaction with UNC-37 and is necessary for rescue of *mls-1* mutants, we hypothesize MLS-1 requires UNC-37 to specify uterine muscle fate. Therefore *unc-37* loss-of-function animals should phenocopy *mls-1* mutants. To test this hypothesis, we performed *unc-37(RNAi)* on *egl-15::gfp* animals. These RNAi affected animals displayed *egl-15::gfp* expression in the four vm1 cells but also in up to four additional cells (Figure 4A, Table 2). To more directly examine the effect on uterine muscle specification we repeated *unc-37(RNAi)* experiments on animals with an integrated *rgs-2::gfp* transgene. In wild-type adults *rgs-2::gfp* is expressed in the pharynx, the ventral nerve cord, lumbar ganglia, and uterine muscles [14], [25]. *unc-37(RNAi)* greatly reduced the number of and intensity of *rgs-2::gfp* expressing uterine muscles, while having no effect on *rgs-2::gfp* expression in other tissues (Figure 4B, Table 2). Note that the morphology of uterine muscles made it much more difficult to count *rgs-2::gfp* expressing cells than *egl-15::gfp* expressing vulval muscles, but nearly half the *unc-37(RNAi)* animals we examined had no *rgs-2::gfp* expressing uterine muscles.

**Table 2:** Vulval and uterine muscle pattern in *unc-37(RNAi)* animals.

Genotype^1^	Normal Sex Muscle Pattern^2^
*egl-15::gfp*	86% (n = 44)
*egl-15::gfp; L4440(RNAi)* 3	100% (n = 36)
*egl-15::gfp; unc-37(RNAi)*	11% (n = 131)
*rgs-2::gfp*	100% (n = 19)
*rgs-2::gfp; L4440(RNAi)* 3	100% (n = 46)
*rgs-2::gfp; unc-37(RNAi)*	51% (n = 41)
*egl-15::gfp; unc-37(wd17wd21)*	19% (n = 59)

Expression of *egl-15::gfp* and *rgs-2::gfp* were used to score vm1 muscles and uterine muscles, respectively. *egl-15::gfp* expression is occasionally mosaic in wild-type and mutant animals. Animals containing four *egl-15::gfp* expressing vm1 vulval muscles were scored as wild type. It was difficult to count *rgs-2::gfp* expressing uterine muscles, so only animals with no *rgs-2::gfp* expression in the uterine muscles were scored as mutant.

1 *ayIs2[egl-15::gfp]* or *vsIs5[rgs-2::gfp]* animals were used.

2 Refers to animals with four cells expressing GFP (*egl-15::gfp*) or any GFP expression around the vulva (*rgs-2::gfp*).

3 L4440 is the RNAi feeding vector containing no insert [40].

We next examined *egl-15::gfp* expression in two *unc-37* mutants: *unc-37(e262)* and *unc-37(wd17wd21)*. *unc-37(e262)* is a hypomorphic allele that can be maintained as a homozygous strain, and it encodes a missense mutation affecting a conserved residue in the WD repeat domain that specifically affects UNC-37 function in VA motor neurons [17]. In contrast *unc-37(wd17wd21)* is a putative null allele containing a splice acceptor site mutation upstream of the WD repeats [17]. *unc-37(wd17wd21)* mutants exhibit a maternal effect embryonic lethal phenotype, but *unc-37(wd17wd21)* homozygotes produced from heterozygous mutant hermaphrodites grow to adulthood and exhibit uncoordinated and protruding vulva phenotypes. Most *unc-37(e262)* mutants exhibited a wild-type pattern of *egl-15::gfp* expression, but approximately 5% of these animals expressed *egl-15::gfp* in extra cells. A much higher percentage of *unc-37(wd17wd21)* adult animals (81%, n = 59) contained supernumerary *egl-15::gfp* expressing vulval muscles. In both cases GFP-expressing cells were near the vulva similar to *mls-1(cc569)* animals (Figure 4A), but in other cases they were located in the posterior body wall muscle quadrants (Figure S2). A similar localization of *egl-15::gfp* expressing cells has previously been observed in *mls-2* mutants [26]. This phenotype may be significant as MLS-2 is predicted to have a high scoring eh1 motif [12], and it suggests that *unc-37(e262)* may affect the activity of both MLS-1 and MLS-2 in specifying cell fates in the M lineage.

## Discussion

### MLS-1 represses transcription to specify uterine muscle fate

MLS-1 is a selector gene that is necessary and sufficient to specify uterine muscle development in the M lineage [14]. Here we show that MLS-1 interacts with the UNC-37/Groucho co-repressor, and that MLS-1 function in *C. elegans* depends on this interaction. These results indicate that MLS-1 specifies uterine muscle fate at least in part by repressing target gene transcription.

What types of genes does MLS-1 regulate? We suggest that MLS-1 represses expression of regulatory genes that themselves encode inhibitors of uterine muscle development and activators of vulval muscle development. This model is consistent with previous observations that *mls-1* loss-of-function results in a transformation of uterine muscle to vulval muscle, whereas ectopic *mls-1* expression results in production of ectopic uterine muscles [14]. Analogous negative regulatory pathways have been suggested for specification of body wall muscle and coelomocyte cell fates elsewhere in the M lineage [27], [28]. The MLS-1 targets must be expressed in the M lineage outside of the descendants of the SMs, because widespread expression of *mls-1* in the M lineage can convert many M lineage cells to a uterine muscle fate [14]. We do not yet know of any direct targets of MLS-1. The *egl-15* promoter is active in the supernumerary vulval muscles in *mls-1* mutants, and we asked if it might be directly repressed by MLS-1. However mutation of predicted T-box binding sites in this promoter did not lead to expanded expression *egl-15::gfp* reporter (R. Miller and P. Okkema, unpublished), suggesting MLS-1 regulation of the *egl-15* promoter is indirect.

MLS-1 may also function with UNC-37/Groucho in other MLS-1 expressing cells. When expressed with its own promoter, MLS-1 interacts with UNC-37 in BiFC assays in the vm2 vulval muscles and one to two intestinal/enteric muscles. The function of *mls-1* in these cell types is unknown [14], so we cannot determine if interaction with UNC-37 is necessary for MLS-1, but we suggest that MLS-1 may similarly function as a Groucho dependent repressor in these cells.

In addition to MLS-1, UNC-37/Groucho likely interacts with other factors that are involved in vulval development or that are expressed in the M-lineage. *unc-37(RNAi)* and some mutant alleles produce a protruding vulva (Pvl) phenotype that is not observed in *mls-1* mutants [29]. Likewise, we also found *egl-15::gfp* expressing cells that looked like body wall muscles in *unc-37(e262)* and more frequently in *unc-37(wd17wd21)* mutants, while we never observed this phenotype in *mls-1* mutants. At least two other transcription factors expressed in the M lineage contain high scoring eh1 motifs (CEH-24 and MLS-2 see [12]), and we suggest that these and other factors expressed in the M lineage function as Groucho-dependent repressors.

### *mls-1^FSI→ASA^* may be a gain-of-function mutation

Our results suggest that mutation of the MLS-1 eh1 motif creates a gain-of-function protein that is deleterious. It was much more difficult to generate transgenic lines expressing *mls-1^FSI→ASA^* than lines expressing wild-type *mls-1* using either the *mls-1* promoter or a heat-inducible promoter, and we were only able to generate transgenic lines expressing *mls-1^FSI→ASA^* with arrays containing a low concentration of the expression vector. MLS-1^FSI→ASA^ contains an intact T-box, and, because many T-box factors bind similar sequences, MLS-1^FSI→ASA^ could recognize binding sites for wild-type MLS-1 or for other T-box factors. It is unlikely that *mls-1^FSI→ASA^* is simply a dominant negative mutation that interferes with wild-type *mls-1* function, because *mls-1* null mutants are viable and healthy [14]. Instead, we suggest that MLS-1^FSI→ASA^ interferes with function of other T-box factors that are required for viability. Alternatively, mutation of the eh1 motif may allow MLS-1 to function as a transcriptional activator and inappropriately activate T-box target genes. Groucho has been shown to convert a variety of transcriptional activators to repressors, including several T-box factors [7]–[10].

### Relationship to T-box factors in other species

MLS-1 is a member of the Tbx1 sub-family, which includes the mammalian proteins Tbx1, Tbx15, Tbx18, Tbx20, and Tbx22. Like MLS-1, each of these proteins has been shown to repress transcription, and there is evidence that these proteins interact either directly or indirectly with Gro/TLE proteins [8]–[12]. Thus this T-box sub-family shares a common mechanism for transcriptional repression in different animal phyla.

Among the mammalian T-box genes, MLS-1 is most closely related to Tbx15, Tbx18 and Tbx22. These genes have diverse functions in mesoderm development, but recently described functions for Tbx18 in smooth muscle development may be most closely related to the function of MLS-1. Tbx18 is expressed in the developing urogenital ridge where it is necessary for development of the ureter smooth muscle [30], [31]. Likewise Tbx18 is also expressed in endocardial cells that contribute to the coronary smooth muscles, although its function in this tissue has not yet been characterized [32]. The *C. elegans* muscle types expressing MLS-1 share both structural and functional similarities with mammalian smooth muscles. *C. elegans* uterine muscles are non-striated with loosely organized myofilaments arranged circumferentially around the uterus, and contractions of these muscles help move embryos through the uterus toward the vulva [14], [15]. Ultrastructurally, the uterine muscles contain thin filaments attached to the uterine basal lamina at randomly arranged points, which is similar to the organization found in smooth muscles [33]. Together, these observations suggest the interesting possibility that Tbx18 and MLS-1 share conserved function in smooth muscle development.

## Materials and Methods

### Strains and plasmids

*C. elegans* were grown under standard conditions and were raised at 20°C unless otherwise noted [34]. The following strains were used: NH2447 *ayIs2 [egl-15::gfp] IV* [35]; PD4285 *mls-1(cc569) I; ayIs2 [egl-15::gfp] IV* [14]; LX354 *lin-15(n765ts); vsIs5 [rgs-2::gfp; lin-15(+)]* [25]; CB262 *unc-37(e262) I* [36], NC93 *unc-37(wd17wd21)/dpy-14(e188) I* [17], OK0675 *unc-37(e262) I; ayIs2 [egl-15::gfp] IV*, and OK0787 *unc-37(wd17wd21)/dpy-14(e188) I; ayIs2 [egl-15::gfp] IV*. The following transgenic strains were constructed for this work.

#### *mls-1(cc569)* rescue lines

Transgenic lines were constructed by injecting PD4825 with plasmids containing either an *mls-1(+)* genomic fragment (pSAK244.13, provided by A. Fire, Stanford) or *mls-1^FSI→ASA^* (pOK257.02) at 2.5 ng/µl and pRF4 at 100 ng/µl [37]: OK0720 *mls-1(cc569); ayIs2; cuEx575[mls-1(+)]*, OK0721 *mls-1(cc569); ayIs2; cuEx576[mls-1(+)]*, OK0722 *mls-1(cc569); ayIs2; cuEx577[mls-1(+)]*, OK0723 *mls-1(cc569); ayIs2; cuEx578[mls-1(+)]*, OK0724 *mls-1(cc569); ayIs2; cuEx579[mls-1(+)]*, OK0725 *mls-1(cc569); ayIs2; cuEx580[mls-1(+)]*, OK0683 *mls-1(cc569); ayIs2; cuEx568[mls-1^FSI→ASA^]*, OK0745 *mls-1(cc569); ayIs2; cuEx596[mls-1^FSI→ASA^]*, OK0746 *mls-1(cc569); ayIs2; cuEx597[mls-1^FSI→ASA^]*, OK0747 *mls-1(cc569); ayIs2; cuEx598[mls-1^FSI→ASA^]*, OK0748 *mls-1(cc569); ayIs2; cuEx599[mls-1^FSI→ASA^]*, OK0749 *mls-1(cc569); ayIs2; cuEx600[mls-1^FSI→ASA^]*, OK0750 *mls-1(cc569); ayIs2; cuEx601[mls-1^FSI→ASA^]*.

#### BiFC lines

Transgenic lines were constructed by injecting N2 with pRF4 at 100 ng/µl and the BiFC plasmids pCE-BiFC-VN173, pCE-BiFC-VC155, pOK257.05 (*mls-1::vn173*), pOK257.06 (*unc-37::vc155*), pOK258.06 (*mls-1^FSI→ASA^::VN173*), pOK263.03 (genomic *mls-1::VN173*), or pOK266.02 (genomic *unc-37::VC155*) at 15 ng/µl: OK0708 *cuEx566[VN173+VC155]*, OK0689 *cuEx573[mls-1::vn173+unc-37::vc155]*, OK0690 *cuEx574[mls-1::vn173+unc-37::vc155]*, OK0691 *cuEx575[mls-1::vn173+unc-37::vc155]*, OK0709 *cuEx567[mls-1^FSI→ASA^::VN173+unc-37::vc155]*, OK0710 *cuEx568[mls-1^FSI→ASA^::VN173+unc-37::vc155]*, OK0742 *cuEx593[genomic mls-1::VN173+genomic unc-37::VC155]*, OK0743 *cuEx594[genomic mls-1::VN173+genomic unc-37::VC155]*, OK0744 *cuEx595[genomic mls-1::VN173+genomic unc-37::VC155]*.

### Bimolecular fluorescence complementation (BiFC)

*mls-1* (Open Biosystems) and *unc-37* (yk727f10 provided by Y. Kohara, National Institute of Genetics, Japan) cDNA were cloned into pCE-BiFC-VN173 and pCE-BiFC-VC155 plasmids (provided by Chang-Deng Hu, Purdue) to generate pOK257.05 (*mls-1::vn173*) and pOK257.06 (*unc-37::vc155*). *mls-1^FSI→ASA^* cDNA was generated by site directed mutagenesis of *mls-1* cDNA (Quikchange II XL, Stratagene), and cloned into pCE-BiFC-VN173 producing pOK258.06 (*mls-1^FSI→ASA^::vn173*). Transgenic adults were picked to OP50 seeded plates, heat shocked for one hour at 33°C as described in Results, allowed to recover for one hour at 20°C, and examined for BiFC signal at 63× magnification.

Genomic *mls-1::vn173* and *unc-37::vc155* constructs, with gene expression under the control of their respective endogenous promoters, were produced by PCR of promoter+gene fragments using pSAK244.13 and N2 genomic DNA templates, respectively. *hsp-16.41* promoter fragments were removed from pCE-BiFC-VN173 and pCE-BiFC-VC155 and replaced with these *mls-1* and *unc-37* promoter+gene cassettes generating pOK263.03 and pOK266.02. Transgenic animals from various stages (L1 to Adult) were examined at 63× for BiFC signal.

### Yeast two-hybrid assays

Yeast two-hybrid assays were performed as previously described [38]. The *mls-1* bait plasmid (pOK248.03) was constructed by inserting the full-length *mls-1* cDNA in to pLexA-NLS, and the *unc-37* prey plasmid was previously isolated from the pACT-RB1 cDNA library (provided by R. Barstead).

### *mls-1(cc569)* rescue assay

*mls-1(cc569); ayIs2* strains with mls-1(+) or *mls-1^FSI→ASA^* extrachromosomal arrays were generated as above. Adult transgenic animals from these lines and animals from NH2447 and PD4285 were examined and scored at 40× and 63× magnification for the number of cells expressing *egl-15::gfp*.

### *unc-37* loss of function

*unc-37* cDNA was cloned into the L4440 (double T7 promoter) vector and the resulting plasmid (pOK247.03) was used to transform HT115(DE3) *E. coli* cells. *unc-37* “feeding” RNAi was performed as before with some modifications [39], [40]. Plates with large numbers of *ayIs2 [egl-15::gfp] IV* (NH2447) or *lin-15(n765ts) X; vsIs5 [rgs-2::gfp]* (LX354) (provided by M. Koelle, Yale) adults and embryos were bleached and embryos were transferred to unseeded NGM plates and placed at 25°C overnight to allow embryos to hatch and synchronize as L1s. The next day synchronized L1s were transferred to *unc-37(RNAi)* seeded plates and placed at 20°C for 48 hours to 72 hours. Adult animals were examined and scored at 40× and 63× magnification for *egl-15::gfp* or *rgs-2::gfp* (mis)expression. Note that it is only possible to qualitatively evaluate *rgs-2::gfp* expression due to the unusual morphology of uterine muscles.

## Supporting Information

Figure S1 *pmls-1::Venus* expression in enteric and sex muscles of L4s and adults. A) L4 animal showing expression of *pmls-1::Venus* in left intestinal (Int) and anal depressor (AnDep) muscles. B) L4 animal (top) showing expression of *pmls-1::Venus* in vulval muscles (VM). A young adult (bottom) displays *pmls-1::Venus* expression in expanded uterine muscles. The *pmls-1::Venus* plasmid contains bp 1–1308 of cosmid H14A12 (Accession AF025459) cloned into pPD95.79-Venus (kindly provided by D. Byrd and J. Kimble).
(TIF)

Figure S2 Ectopic expression of *egl-15::gfp* in body wall muscle cells of *unc-37(e262)* animals. Image of two *unc-37(e262); ayIs2[egl-15::gfp]* adults shown at 40× and 63× magnification. Arrowheads point to supernumerary vulval muscles. Arrows point to cells with body wall muscle morphology that ectopically express *egl-15::gfp*.
(TIF)
