# Supplementary figures and images for: The Caenorhabditis elegans T-Box Factor MLS-1 Requires Groucho Co-Repressor Interaction for Uterine Muscle Specification
Source: PLoS Genet. 2011 Aug 11;7(8):e1002210. doi: 10.1371/journal.pgen.1002210 (PMC3154951; doi:10.1371/journal.pgen.1002210)

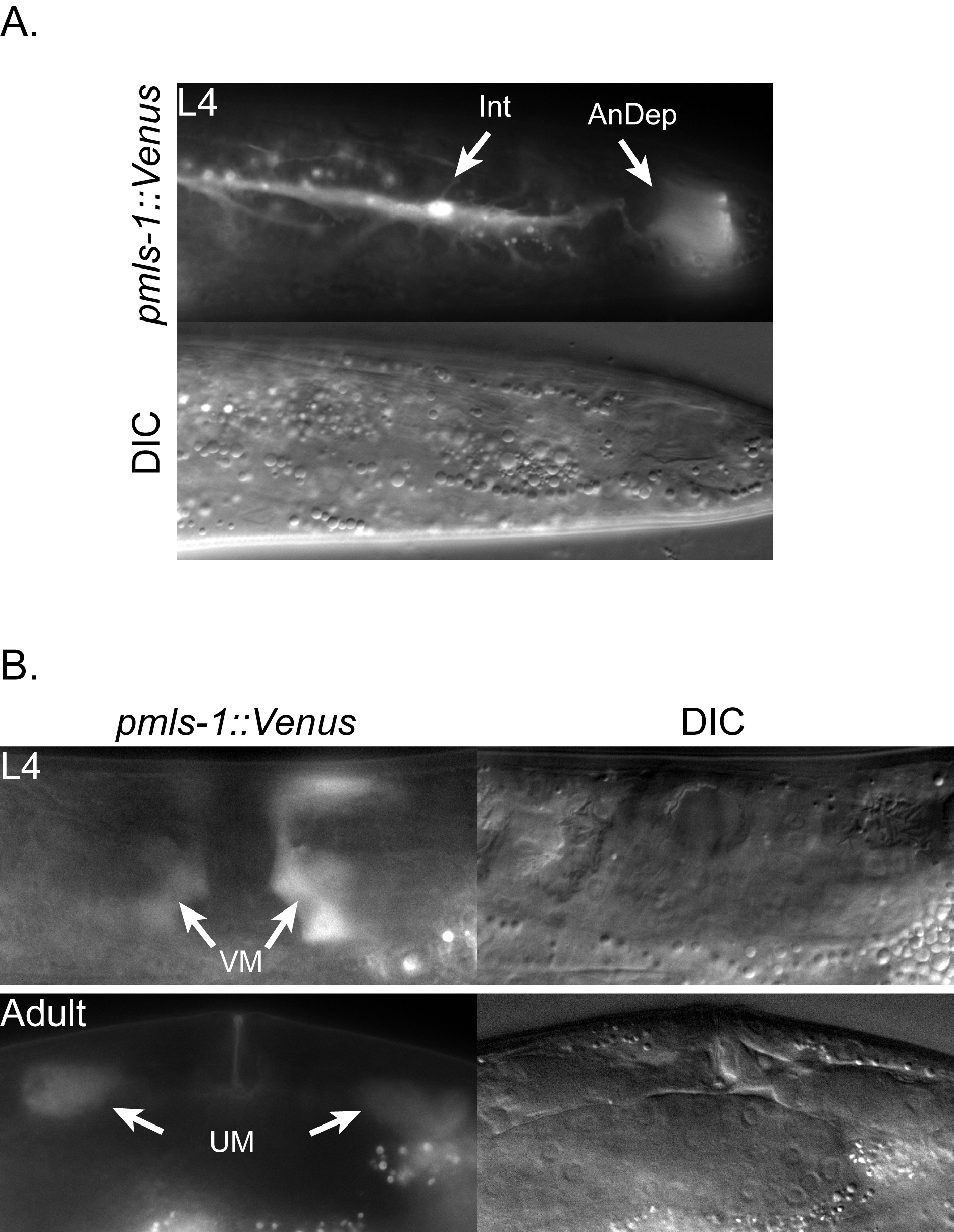

Supplement: Figure S1 — pmls-1::Venus expression in enteric and sex muscles of L4s and adults. A) L4 animal showing expression of pmls-1::Venus in left intestinal (Int) and anal depressor (AnDep) muscles. B) L4 animal (top) showing expression of pmls-1::Venus in vulval muscles (VM). A young adult (bottom) displays pmls-1::Venus expression in expanded uterine muscles. The pmls-1::Venus plasmid contains bp 1–1308 of cosmid H14A12 (Accession AF025459) cloned into pPD95.79-Venus (kindly provided by D. Byrd and J. Kimble). (TIF) [file pgen.1002210.s001.tif]

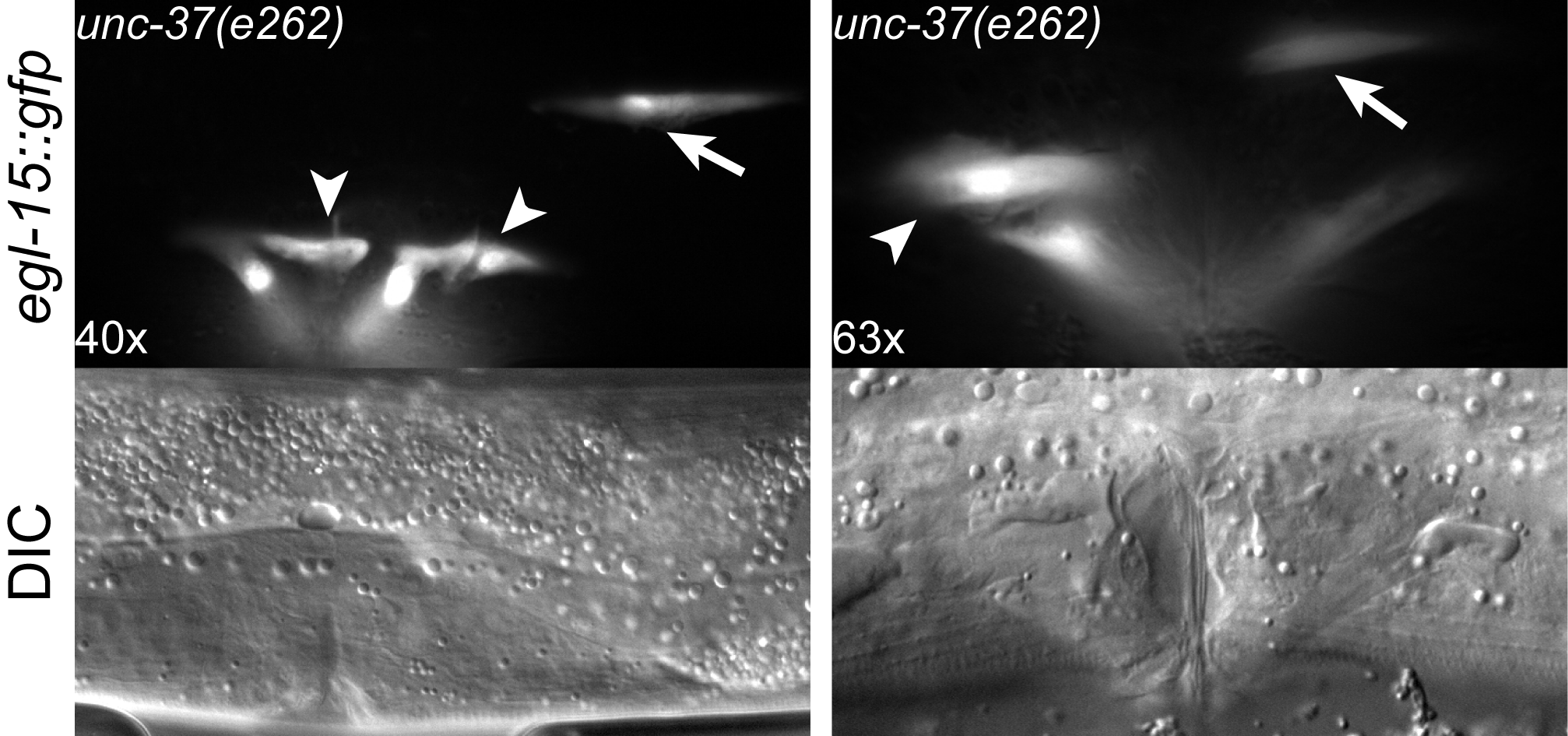

Supplement: Figure S2 — Ectopic expression of egl-15::gfp in body wall muscle cells of unc-37(e262) animals. Image of two unc-37(e262); ayIs2[egl-15::gfp] adults shown at 40× and 63× magnification. Arrowheads point to supernumerary vulval muscles. Arrows point to cells with body wall muscle morphology that ectopically express egl-15::gfp. (TIF) [file pgen.1002210.s002.tif]
